# Relevance of the NLRP3 Inflammasome in the Pathogenesis of Chronic Liver Disease

**DOI:** 10.3389/fimmu.2017.01728

**Published:** 2017-12-12

**Authors:** Xiaoqin Wu, Lei Dong, Xianhe Lin, Jun Li

**Affiliations:** ^1^Department of Cardiology, First Affiliated Hospital of Anhui Medical University, Hefei, China; ^2^Department of Pediatrics, Division of Hematology/Oncology, Aflac Cancer and Blood Disorders Center, Children’s Healthcare of Atlanta, Emory University School of Medicine, Atlanta, GA, United States; ^3^School of Pharmacy, Institute for Liver Diseases of Anhui Medical University, ILDAMU, Key Laboratory of Anti-Inflammatory and Immune Medicine, Anhui Medical University, Hefei, China; ^4^School of Life Science, Beijing Institute of Technology, Beijing, China

**Keywords:** inflammasome, inflammation, viral hepatitis, non-alcoholic steatohepatitis, alcoholic liver disease

## Abstract

Inflammation is a common characteristic of chronic liver disease (CLD). Inflammasomes are multiprotein complexes that can sense and recognize various exogenous and endogenous danger signals, eventually activating interleukin (IL)-1β and IL-18. The sensor component of the inflammasome system is a nucleotide-binding oligomerization domain (NOD)-like receptors (NLRs). The NLRs family pyrin domain containing 3 (NLRP3) inflammasome has been involved in the initiation and progression of CLD. However, the molecular mechanisms by which it triggers liver inflammation and damage remain unclear. Here, we focus on recent advances on the potential role of NLRP3 inflammasome activation in the progression of CLD, including viral hepatitis, non-alcoholic steatohepatitis and alcoholic liver disease, and in particular, its ability to alleviate liver inflammation in animal models. Additionally, we also discuss various pharmacological inhibitors identifying the NLRP3 inflammasome signaling cascade as novel therapeutic targets in the treatment of CLD. In summary, this review summarizes the relevance of the NLRP3 inflammasome in the initiation and progression of CLD, and provides critical targets to suppress the development of CLD in clinical management.

## Introduction

**Figure 1:**
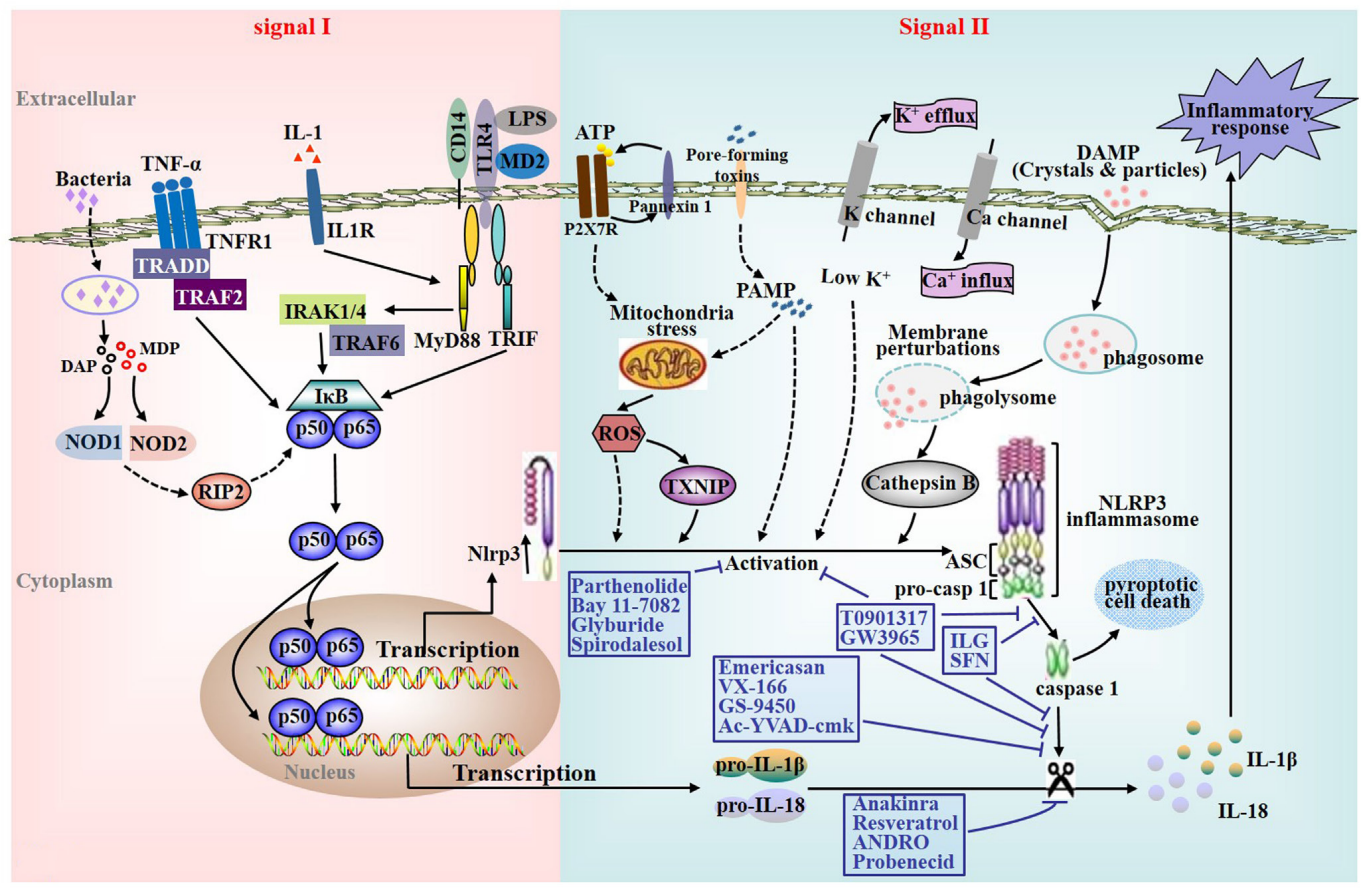
Nucleotide-binding oligomerization domain-like receptors family pyrin domain containing 3 (NLRP3) inflammasome activation and characterization of various pharmacological inhibitors of the NLRP3 inflammasome signaling cascade. Effective activation of the NLRP3 inflammasome requires a priming signal prior to or coincident with a secondary NLRP3-specific activating signal. First, NLRP3 inflammasome priming or initiating is accomplished by nuclear factor kappa B (NF-κB)-activating receptors including Toll-like receptors (TLRs), interleukin (IL)-1 receptor, tumor necrosis factor receptor, and the cytosolic pattern recognition receptor nucleotide-binding oligomerization domain 2 (signal I). Subsequently, the second signaling is provided by one of various agonists that triggers NLRP3-specific activation, assembling of the NLRP3 inflammasome complex, and ultimately culminates in the activation of casp1 as well as the secretion of mature IL-1β and IL-18 (signal II). So far, a variety of triggers with different properties have been shown for NLRP3 inflammasome activation. The diverse group of activators includes crystals and particles such as alum, silica, asbestos, monosodium urate that requires phagocytosis for activation, adenosine triphosphate (ATP) acting *via* its cell surface purinergic P2X7 receptor (ROS), and pore-forming toxins such as nigericin.

Inflammasomes are cytoplasmic multiprotein complexes responsible for caspase-1 (casp1) activation with the subsequent production of the cytokines interleukin (IL)-1β and IL-18, and the initiation of the inflammatory cell death termed pyroptosis (Figure 1). These complexes consist of one of several nucleotide-binding oligomerization domain (NOD)-like receptors (NLRs) and the pyrin and HIN200 family (1). Inflammasomes are major contributors to inflammation, which can sense both endogenous and exogenous danger signals through intracellular NLRs (2). There are four main prototypes of inflammasomes including NLR family pyrin domain containing 1 (NLRP1), NLRP3, NLRC4, and absent in melanoma 2 (3). So far, the NLRP3 inflammasome is the most extensively studied (4). The NLRP3 inflammasome is typically composed of an inflammasome sensor NLRP3, the adaptor molecular apoptosis-associated speck-like protein containing a casp-recruitment domain (ASC) and the precursor pro-casp1 (Figure 1) (4, 5). Previous studies on the NLRP3 inflammasome were primarily focused on innate immunity and showed that it can recognize a variety of structurally dissimilar agonists, such as pathogen-associated molecular patterns, pore-forming toxins, environmental irritants and damage-associated molecular patterns (6, 7). Upon activation, assembly of NLRP3 inflammasome components resulted in the activation of casp1, which further triggers the maturation and secretion of proinflammatory cytokines including IL-1β and IL-18 (2, 8–10). Furthermore, several studies have reported that NLRP3 inflammasome activation is also responsible for the development of chronic inflammation-related diseases, especially chronic liver disease (CLD).

Hepatitis viruses are the main pathogens in the development of CLD. Liver inflammation contributes to the development of infection-induced liver fibrosis (LF), cirrhosis, and hepatocellular carcinoma (HCC). Nevertheless, the processes that initiate liver inflammation by hepatotropic viruses, such as hepatitis virus B (HBV) and hepatitis virus C (HCV), are not well defined. Recently, accumulating evidence pointed out the pivotal role of the NLRP3 inflammasome in the development of CLD. Negash et al. demonstrated that HCV infection induced a potassium efflux, which activated the NLRP3 inflammasome in Kupffer cells (KCs) for IL-1β production. In turn, IL-1β secretion enhanced chemokine, proinflammatory cytokine, and immune-regulatory gene expression networks, which are associated with chronic hepatitis C (CHC) severity (11). Additionally, another study showed the NLRP3 inflammasome could contribute to LF as well in the development of nonalcoholic steatohepatitis (NASH) in mice (12). They found that NLRP3 inflammasome activation resulted in severe liver inflammation, hepatocyte pyroptosis, and hepatic stellate cell (HSC) activation with collagen deposition (13). Therefore, NLRP3 inflammasome activation is involved in the pathogenesis of CLD. However, the mechanisms underlying NLRP3 inflammasome-mediated hepatic inflammation and damage by hepatitis virus, alcohol and high-fat fed are still not fully understood. Herein, this review highlights the current knowledge of the functions of the NLRP3 inflammasome during CLD development and its ability to fine-tune liver inflammation. Additionally, we also discuss inhibitors identifying the NLRP3 inflammasome signaling cascade as novel therapeutic targets in the treatment of CLD.

## NLRP3 Inflammasome Activation

Understanding NLRP3 inflammasome activation is a crucial step given its potential role in multiple aspects of CLD (14–16). The expression of NLRP3 itself appears to be the limiting factor for the activation of the NLRP3 inflammasome (17). Current studies suggest that its effective activation requires a priming signal (signal I) prior to or coincident with a secondary NLRP3-specific activating signal (signal II) (18, 19). First of all, NLRP3 inflammasome priming or initiating (signal I) is accomplished by nuclear factor kappa B (NF-κB)-activating receptors, such as tumor necrosis factor receptor, toll-like receptors (TLRs), IL-1 receptor, and the cytosolic pattern recognition receptor NOD2. Due to the fact that the endogenous levels of NLRP3 are inadequate for efficient inflammasome activation and pro-IL-1β is not constitutively expressed, the activation as well as nuclear translocation of NF-κB is crucial for upregulating the transcription of NLRP3, casp1, and pro-IL-1β. Furthermore, signal I leads to posttranslational regulation of inflammasome components including NLRP3 deubiquitination as well as ASC ubiquitination and phosphorylation (Figure 1) (20, 21).

Subsequently, the second signaling is provided by one of various agonists that triggers NLRP3-specific activation, assembling of inflammasome complex, and ultimately results in the activation of casp1 and the production of mature IL-1β and IL-18 (signal II) (22, 23). In addition, the activation of casp1 induces the production of IL-1α as well (21). So far, a variety of triggers have been shown for the activation of the NLRP3 inflammasome. The diverse group of activators includes crystals and particles such as asbestos, silica, alum, crystalline monosodium urate that requires phagocytosis for inflammasome activation, adenosine triphosphate (ATP) acting *via* purinergic P2X7 receptor (ROS), and pore-forming toxins such as nigericin. Although the exact molecular mechanisms of inflammasome activation by various activators are unknown, several intriguing models have been developed to illuminate the signal II of the activation process (4). In brief, all current models assume that the NLRP3 inflammasome does not directly interplay with exogenous stimuli, which is accordance with its ability to sense a variety of pathogens. Since these agonists are structurally dissimilar and further act upon the cell in different ways, the direct binding of a ligand to the NLRP3 inflammasome most likely occur downstream from these irrelevant upstream activators.

## The NLRP3 Inflammasome in Viral Hepatitis

### Role of NLPR3 in Patients with Chronic Hepatitis B (CHB)

The most common viral hepatitis, hepatitis B and hepatitis C, which is typically related to chronic viral infection. Among them, HBV is a non-cytopathic double-stranded, hepatotropic DNA virus. Currently, there are estimated to be 2 billion people who have been infected with HBV, and an estimated 400 million people worldwide are CHB carries (24, 25).

Askari et al. showed that the expression of the NLRP3 inflammasome transcripts was not significantly different among CHB patients, regardless of the HBV-DNA copy numbers, suggesting that HBV replication is not associated with altered expression of NLRP3. Meanwhile, only alkaline phosphatase level had a poor positive correlation with NLRP3, whereas serum levels of aspartate aminotransferase (AST), alanine aminotransferase (ALT), and total and direct bilirubin were not associated with NLRP3 expression. Therefore, these results indicate that NLRP3 expression might be associated with CHB complications such as liver damage (26). However, there is currently no direct evidence supporting the function of the NLRP3 inflammasome in patients with CHB. Interestingly, a recent study by Yu et al. showed that HBeAg, not HBsAg, could suppress LPS-induced the activation of the NLRP3 inflammasome and casp1 as well as IL-1β maturation and production by inhibiting NF-κB phosphorylation and ROS production (27), which suggests a more enhanced understanding of the interaction between the NLRP3 inflammasome and HBV. Further studies have to be performed to investigate the potential role of the NLRP3 inflammasome in the pathogenesis of CHB.

### Role of NLPR3 in Patients with CHC

Hepatitis virus C is known a hepatotropic and enveloped virus, which carries a positive-sense single-stranded RNA genome with approximately 9.6 kb nucleotides in length, and chronically infects approximately 3% of people worldwide (28). HCV is the most common human bloodborne virus, of which acute infection have a higher risk of progressing to chronic infection (29). Generally, virus infects hepatocytes to impart chronic hepatic inflammation and further progressive liver injure driving LF, cirrhosis and even HCC (30). Chronic inflammation is the basis of HCV-mediated liver damage. Understanding the molecular mechanisms underlying liver inflammation induced by HCV is critical for establishing several novel approaches to reduce HCV-induced liver diseases. Burdette et al. found the activation of the NLRP3 inflammasome in JFH-1 (human hepatoma cell line) infected with HCV (31). Importantly, they also demonstrated that HCV could activate IL-1β *via* casp1-inflammasome complex. Furthermore, HCV-induced production of IL-1β was reduced by inhibiting the assembling of the inflammasome complex using a small interfering RNA approach against NLRP3, ASC or casp1 (31). These findings provide a new explanation for the mechanisms of chronic HCV infection in inflammatory processes *in vitro*. However, the activation of the NLRP3 inflammasome in hepatocytes may not be the main cell population of proinflammatory cytokine IL-1β-producing in patients with CHC. The expression of the inflammasome in various primary hepatic cell subpopulations during experimental liver fibrogenesis was studied. Boaru et al. found the expression of NLRP3 inflammasome components was prominent in liver sinusoidal endothelial cells and KCs in experimental mouse models of inflammatory and fibrotic liver disease, moderate in cultured HSCs and periportal myofibroblasts, while was almost absent in primary hepatocytes (32). In accordance with this work, Negash et al. showed that the serum levels of IL-1β were significantly increased in patients with CHC and KCs were the major IL-1β-producing cell population during HCV infection. Furthermore, they showed that exposure of THP-1 cells to HCV induced IL-1β production and secretion through MyD88-dependent TLR7 signaling and the NLRP3 inflammasome pathway to induce IL-1β processing and secretion. Additionally, using RNA sequencing analysis of liver biopsies in HCV-infected patient, they revealed that HCV triggering of these signaling pathways upregulated proinflammatory cytokine and immune-regulatory gene expression networks (11). In summary, these works provide multiple lines of evidence to demonstrate that IL-1β maturation and secretion by KCs confers hepatic inflammation *via* HCV-induced inflammasome signaling through a potassium efflux (33). In addition, Chen et al. reported that HCV-induced NLRP3 inflammasome activation and IL-1β secretion required ROS production (34). Besides, McRae’s group found that HCV could exploit the NLRP3 inflammasome to activate the sterol regulatory element-binding proteins and host lipid metabolism, resulting in the pathogenesis associated with CHC (35).

Taken together, these observations suggest that inflammasome complex components and/or IL-1β activity may become novel therapeutic targets of reducing hepatic inflammation and mitigating CHC. Although HCV-infected patients have been reported to have a poor response to interferon (IFN) therapy (36). IFN could inhibit the activity of the NLRP3 inflammasome signaling through suppressing casp1-dependent IL-1β maturation (37). However, IFN is well known in immunomodulatory response against viral infections, endogenous IFN production causes innate immune tolerance that may contribute to the failure of IFN use in HCV-infected patients (36, 38). Therefore, effective IFN-based therapy for CHC reduces hepatic inflammation (39), which has been considered to minimize side effects including immune tolerance, induced by endogenous IFN, even though this complex process involves IFN/IL-1β interactions (40). Strategies to inhibit NLRP3 inflammasome complex components or IL-1β activity provide therapeutic actions to alleviate liver inflammation and mitigate CHC, particularly where antiviral agents have failed.

## The NLRP3 Inflammasome in NASH

Nonalcoholic steatohepatitis is a serious liver condition, which is closely related to insulin resistance and obesity. Although the mRNA levels of NLRP3 inflammasome components were upregulated in the liver of early steatosis in animal models, the NLRP3 inflammasome was not shown activated. However, when non-alcoholic fatty liver disease (NAFLD) advanced to NASH, the gene expression of NLRP3 inflammasome components was significantly increased and the inflammasome was fully activated in the liver (13, 41). Consistent with animal data, there was a significant increase in the mRNA expression of inflammasome components (NLRP3, ASC, casp1, pro-IL-1β, and pro-IL-18) in the liver of NASH patients (12, 41). Further study by Wree et al. showed NLRP3 inflammasome activation could contribute to LF in the development of NASH in mice on choline-deficient amino acid-defined feeding (12). Furthermore, in patients with NASH, the mRNA expression of pro-IL-1β correlated with levels of Colla1, a key profibrogenic gene (12). Therefore, the NLRP3 inflammasome was associated with liver disease progression from benign hepatic steatosis to NASH.

Given the potential role of NLRP3 inflammasome activation in NASH, characterizing the specificity of its activation in the liver is critical. NLRP3 inflammasome activation in different cell types has been addressed. Initially, the presence and functions of the NLRP3 inflammasome have been shown in liver immune cells (42). Miura et al. found that KCs produced IL-1β (43). In another report, Dixon et al. demonstrated that KCs was a key cellular source of marked casp1 activation in the liver of methionine-choline-deficient (MCD)-fed mice (44). Further study by Olteanu et al. uncovered that selective deficiency of IL-1α in KCs reduced hepatic inflammation and the mRNA levels of various proinflammatory cytokines including TNFα, IL-1α, IL-1β, IL-6, and serum amyloid A1 in the liver, which may protect against steatohepatitis development (45). However, in this model, there is no direct evidence to show that IL-1α production in KCs is inflammsome dependent. Stimuli such as viral infection, cholesterol crystals or dying cells could elicit IL-1α production independently of inflammasome activation (46–49). Therefore, we propose that IL-1α production in KCs is either inflammasome/casp1 dependent or not in this study. In addition to liver immune cells, there was accumulating evidence that the NLRP3 inflammasome was functionally activated in parenchymal cells including hepatocytes (41). Kamari et al. found that IL-1α derived from hepatocyte, not from recruited bone marrow-derived cells (BMDC), was required for steatohepatitis development (50). The contribution of the NLRP3 inflammasome in hepatocytes was further supported by Csak et al., who demonstrated that saturated fatty acids (FAs) represented an endogenous danger signal as a first hit, triggered NLRP3 inflammasome activation in mouse models of MCD diet-induced and high-fat diet-induced NASH, and ultimately induced sensitization to a second hit with LPS for the production of cytokine IL-1β in mouse hepatocytes (41). Interestingly, hepatocytes treated with saturated FAs could release danger signals that upregulated and activated the NLRP3 inflammasome in liver mononuclear cells (41), suggesting that NLRP3 inflammasome activation may involve the coordinated interaction between immune cells and hepatocytes. This work was further supported by Csak’s group, who also found that both BMDCs and hepatocytes contributed to the activation of NLRP3 inflammasome in a MyD88-dependent manner in MCD-fed mice (51).

To further dissect the cell-specific role of the NLRP3 inflammasome in different cell types, Wree et al. found that global *Nlrp3* mutant resulted in marked hepatocyte pyroptosis, severe inflammation and LF, while myeloid cell restricted mutant mice exhibited a less severe liver phenotype without detectable hepatocyte pyroptosis (13). Although these findings indicate the specific contribution of persistent NLRP3 inflammasome activation in hepatic immune versus parenchymal cells to liver pathology *in vivo*, it is better to ascertain the cell-specific effect of the NLRP3 inflammasome in animal models of NASH. Henao-Mejia et al. revealed that in comparison with WT mice, bone marrow-specific NLRP3 and ASC deficiency mice did not show any dramatic difference in MCD-induced NASH severity. On the other hand, NLRP3 knock-in mice specifically in hepatocytes or CD11c^+^ myeloid cells did not exhibit any exacerbation of NASH (52). Hence, these observations suggest that the severity of NASH in inflammasome-deficient mice might not depend on inflammasome functions in hepatocytes or myeloid cells. Aberrations in NLRP3 inflammasome functions in alternative cells other than myeloid cells or hepatocytes may be critical determinants of the enhanced NASH progression in inflammasome-deficient mice. Unsurprisingly, the NLRP3 inflammasome in intestinal epithelial cells was found to be a contributor of NASH progression through regulating the configuration of the intestinal microbiota (52). Besides, the NLRP3 inflammasome was present in HSCs as well, which could regulate various biological functions of HSCs, and are required for LF development (53, 54). In brief, NLRP3 inflammasome activation has been described in immune cells and non-immune cells such as hepatocytes, epithelial cells and HSCs, even though different murine models of NASH were employed. Importantly, its activation may involve the cross-talk between these cells.

## The NLRP3 Inflammasome in Alcoholic Liver Disease (ALD)

Alcoholic liver disease is a term that encompasses the liver manifestations of alcohol overconsumption, including alcoholic fatty liver, alcoholic hepatitis, and chronic alcoholic hepatitis with LF and even cirrhosis (55). It is characterized by hepatic lipid accumulation (steatosis) and innate immune activation and inflammation (56). Activation of KCs plays a critical role in the initiation and development of ALD (57, 58). The NLRP3 inflammasome has been found to be activated in KCs of ethanol-fed mice (42). In accordance with this work, Cui et al. showed the increased levels of the NLRP3 inflammasome components NLRP3, ASC and cleaved casp1 and the upregulation of mature IL-1β in KCs of ethanol-fed mice. Importantly, elevated liver expression of inflammasome components (IL-1β, IL-18, casp1) has been found and correlated with liver injury in patients with ALD (59). Further study showed that NLRP3 deficiency resulted in the attenuation of alcoholic steatosis, similarly as KCs depletion, which barely showed increased hepatic natural killer T (NKT) cells numbers and activation (60). However, DeSantis et al. found that NLRP3 deficiency mice exhibited more severe hepatic damage with higher levels of ALT, increased expression of IL-18 and decreased expression of IL-1β, which suggests that the NLRP3 inflammasome is protective during ethanol-induced hepatic damage (61). These different results might be explained by the different animal gender or other experimental conditions and methods. Recent studies have also investigated the critical importance of IL-1 signaling in ALD using casp1^−/−^, ASC^−/−^, or IL-1R^−/−^ knockout mice, indicating that casp1-dependent upregulation of IL-1β expression and signaling pathway regulated by IL-1R1 are pivotal in the initiation and development of ALD (42).

## Pharmacological Inhibitors of the NLRP3 Inflammasome

Given the critical contribution of NLRP3 inflammasome activation in the pathogenesis of CLD, pharmacological inhibitors of this signaling cascade in CLD are currently attracting attention (Figure 1).

On the one hand, some small molecular compounds as novel non-special inhibitors of NLRP3 inflammasome activation for treatment of CLD have been identified. In a previous study, Juliana et al. identified parthenolide, an herbal NF-κB inhibitory compound, and Bay 11-7082, a synthetic IκB kinase-β inhibitory compound, as direct inhibitors of the NLRP3 inflammasome. *In vitro* assays demonstrated that the effect of these two inhibitors on NLRP3 inflammasome activation was partly regulated by inhibiting the ATPase activity of NLRP3. Additional investigations of parthenolide revealed that it also directly suppressed the protease activity of casp1 by alkylation of the p20 subunit (62). Subsequently, Hiroe et al. demonstrated that isoliquiritigenin (ILG), another small molecular compound, significantly suppressed NLRP3-activated ASC oligomerization and reduced casp1 activation and IL-1β secretion mediated by the NLRP3 inflammasome more effectively than parthenolide and glyburide, known inhibitors of the NLRP3 inflammasome. Moreover, they found that ILG downregulated diet-induced casp1 and IL-1β expression from obesity, NASH, adipose tissue inflammation, and insulin resistance (63). Recently, Yang et al. reported that sulforaphane (SFN) restrained HFD-induced NAFLD through decreasing the expression levels of ASC and casp1 mRNA and IL-1β production in the liver (64). Furthermore, two liver X receptors T0901317 and GW3965 were identified to potently inhibit NLRP3 inflammasome activation, which was accomplished by the reduction of inflammasome-associated mtROS production, ASC oligomerization, casp1 cleavage, and IL-1β secretion. Additionally, these agonists suppressed the priming signaling of NLRP3 inflammasome activation (65). Besides, a unique mode of action in inhibiting NLRP3 inflammasome activation was recently described by Zhang et al., who found that spirodalesol repressed NLRP3 inflammasome assembly, while displayed neither inhibition of p65 activation and luciferase activity of NF-κB nor IL-1β mRNA levels in BMDMs, suggesting that spirodalesol selectively blocked signal II but not signal I of NLRP3 inflammasome activation (66).

On the other hand, casp inhibitors are available for the treatment of CLD as well. A potent pan-casp inhibitor, emricasan (also called IDN-6556, PF-03491390), has been found to reduce liver injury and LF in NASH mouse model (67). Importantly, emricasan has been evaluated in human liver diseases. Although there was no significant decrease in viral RNA levels, administration of emricasan for 14 days rapidly and remarkably reduced the expression of ALT and AST in patients infected with HCV without apparent drug-related adverse experiences (68). In accordance with these observations, a randomized, double-blind, parallel-dose, placebo-controlled clinical study in 204 patients with CHC by Shiffman et al. also showed that emricasan dramatically inhibited serum levels of AST and ALT in this population, and even was well tolerated for more than 12 weeks (69). To date, emricasan has been investigated in over 650 subjects in clinical trials, which has been confirmed to show a beneficial effect on serological biomarkers in patients with CHC, regardless of the cause of disease. More importantly, clinical trials also demonstrated that emricasan did not reduce normal levels of casp activity in healthy controls. Besides emricasan, an *in vivo* study by Morrison et al. showed that treatment with Ac-YVAD-cmk, another casp1 inhibitor, blocked NASH development and insulin resistance in male LDLR^−/−^.Leiden mice (70). In line with the observed reductions in NASH development, they also observed the attenuation in the development of LF, as evaluated by hepatic mRNA expression profiles of fibrosis-related genes including Tnfa, Acta2, and Col1a1, and histological quantification of collagen staining (70). Taken together, these findings indicate that a variety of casp inhibitors may be a novel and effective antifibrotic approach to the treatment of NASH, which is consistent with evidence that administration of VX-166, an irreversible pan-casp inhibitor, could decrease α-smooth muscle actin (α-SMA) expression and hepatic mRNA levels of Colla1 in MCD-fed mice (71). Furthermore, Anstee et al. also determined the effects of VX-166 in HFD-induced steatosis and MCD-induced steatohepatitis. They found that VX-166 had no effect on steatosis, while attenuated oxidative stress, ALT levels and liver inflammation, particularly in MCD-fed mice (72). In addition, GS-9450, a selective casp inhibitor, was studied in a phase 2, double-blind, randomized, placebo-controlled clinical trial. Treatment of NASH patients with GS-9450 reduced ALT levels in a dose-dependent manner, whereas slightly reduced cytokeratin-18 fragment levels and AST levels (73). Except from NASH, GS-9450 also has been demonstrated to have hepatoprotective activity in HCV-infected patients (74).

In addition to casp inhibitors, interference with IL-1β signaling in an NLRP3 inflammasome-dependent manner may also exert beneficial effects on the treatment of CLD. Petrasek et al. found that *in vivo* disruption with anakinra, a recombinant IL-1 receptor (IL-1Rα), inhibited IL-1 signaling and significantly ameliorated ethanol-induced hepatic inflammation, hepatic injury, and steatosis (42). Furthermore, a study in HFD-fed mice by Yang et al. showed that resveratrol possessed a great potential as a liver-protective agent, which is accomplished by amelioration of hepatic metaflammation, inhibition of NLRP3 inflammasome activation, and the reduced expression of proinflammatory markers including TNF-α, IL-1, and IL-6 (75). Recently, Cabrera et al. found that andrographolide (ANDRO), a botanical compound, downregulated LPS-induced IL-1β secretion through inflammasome modulation by a NF-κB-dependent mechanism in experimental NASH (76). However, Chachay et al. performed a double-blind, randomized, placebo-controlled clinical trial to evaluate the effects of resveratrol in a small NAFLD patient cohort and found that treatment with resveratrol for 8 weeks did not remarkedly suppress NAFLD progression compared to the placebo control (77). These observations suggest that the potential therapeutic value of IL-1 inhibitors to treat NAFLD still remains unclear.

Since multiple danger signals are involved in NAFLD, interventions of one proinflammatory signaling might be insufficient to ameliorate hepatic inflammation and liver injury. Recently, researchers pay more attentions to tissue sterile danger signals, which are major contributors to NLRP3 inflammasome activation in the initiation and development of CLD (78, 79). Hence, elimination of sterile danger signals might prevent hepatic inflammation and damage. As expected, pharmacological depletion of uric acid with allopurinol significantly protected from ethanol-induced hepatic injury, inflammatory response and steatosis in mice, and additional protection was achieved by probenecid, which inhibits uric acid and impedes ATP-induced P2X7 signaling (80). Gicquel et al. has reviewed that P2X7R blockade might be a novel potential target for the prevention and treatment of fibrotic diseases correlated with inflammatory process (81).

Taken together, these observations provide the characterization of various pharmacological inhibitors of NLRP3 inflammasome activation in CLD (Table 1), thereby supporting the importance of the NLRP3 inflammasome signaling cascade in the development of CLD. Further studies in animal models and patients with CLD are required to evaluate the therapeutic effect of these inhibitors.

**Table 1:** Pharmacological inhibitors of the NLRP3 inflammasome signaling cascade.

Inhibitors	Chemical structure	*In vitro/in vivo* effect	Reference
Parthenolide	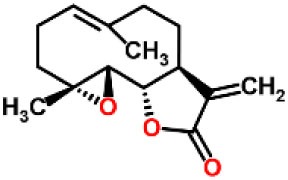	Inhibited the ATPase activity of NLRP3; suppressed the protease activity of caspase-1 by alkylation of the p20 subunit.	(62)
Bay 11-7082	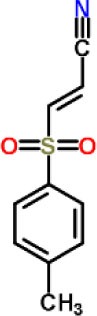	Inhibited the ATPase activity of NLRP3.	(62)
Isoliquiritigenin (ILG)	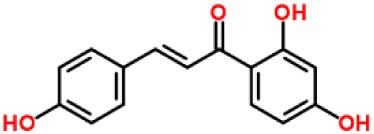	Reduced IL-1β secretion and caspase-1 activation; inhibited diet-induced IL-1β and caspase-1 expression from obesity, NASH, adipose tissue inflammation, and insulin resistance.	(63)
Sulforaphane (SFN)	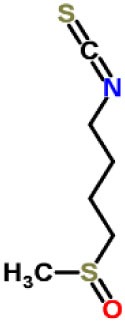	Prevented HFD-induced NAFLD in mice by decreasing the mRNA levels of ASC and caspase-1 and IL-1β production in the liver.	(64)
T0901317	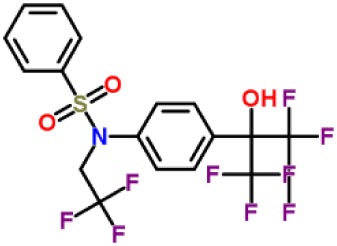	Inhibited NLRP3 inflammasome activation, reduced IL-1β secretion, caspase-1 cleavage and ASC oligomerization, and inflammasome-associated mtROS production; suppressed the priming of inflammasome activation.	(65)
GW3965	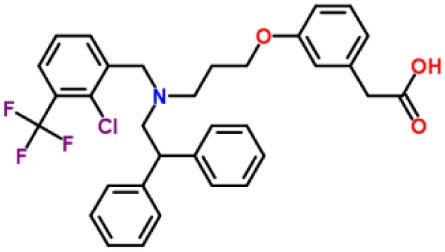	Inhibited NLRP3 inflammasome activation, reduced IL-1β secretion, caspase-1 cleavage and ASC oligomerization, and inflammasome-associated mtROS production; suppressed the priming of inflammasome activation.	(65)
Spirodalesol	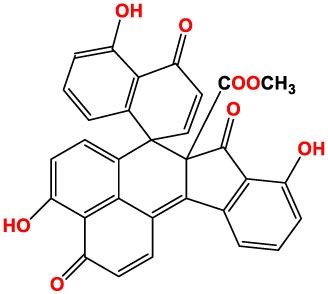	Repressed NLRP3 inflammasome assembly; selectively blocked signal II of NLRP3 inflammasome activation.	(66)
Emricasan (IDN-6556, PF-03491390)	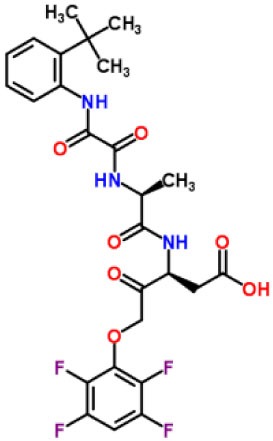	Had a beneficial effect on serological biomarkers in patients with CHC; did not reduce normal levels of caspase activity in healthy controls.	(67–69)
Ac-YVAD-cmk	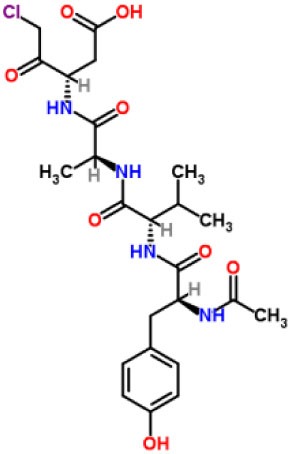	Retarded the development of NASH and insulin resistance in male LDLR^−/−^.Leiden mice; attenuated the development of LF.	(70)
VX-166	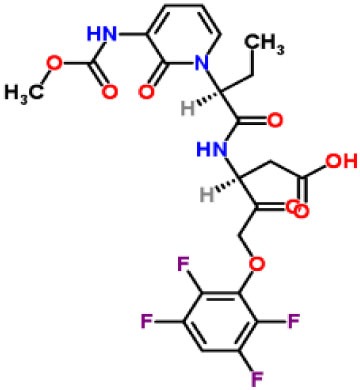	Could decrease α-smooth muscle actin (α-SMA) expression and hepatic mRNA levels of Colla1 in MCD-fed mice; attenuated liver inflammation, ALT levels, and oxidative stress in HF-fed and MCD-fed mice.	(71, 72)
GS-9450	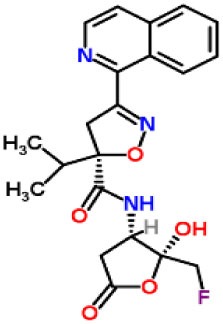	Reduced ALT levels in a dose-dependent manner and slightly reduced cytokeratin-18 fragment levels and AST levels in NASH patients; had hepatoprotective activity in HCV-infected patients.	(73, 74)
Anakinra	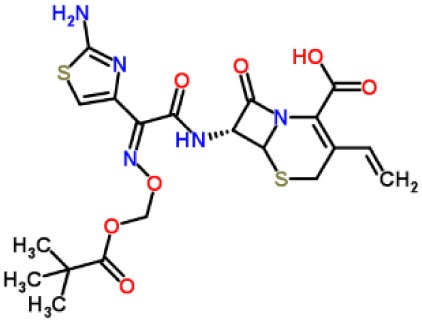	Blocked IL-1 signaling and significantly ameliorated alcohol-induced hepatic inflammation, steatosis, and liver injury.	(42)
Resveratrol	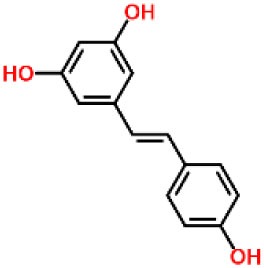	Ameliorated hepatic inflammation, NLRP3 inflammasome activation, and reduced the levels of proinflammatory markers, such as IL-1, IL-6, and TNF-α.	(75)
Andrographolide (ANDRO)	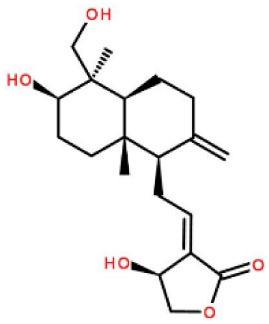	Downregulated LPS-induced IL-1β secretion through inflammasome modulation by a NF-κB-dependent mechanism in experimental NASH.	(76)
Probenecid	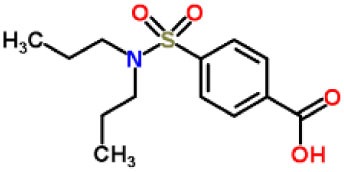	Protected from alcohol-induced inflammatory response, steatosis and hepatic damage in mice.	(80, 81)

## Conclusion

As outlined in this review, there is emerging evidence that NLRP3 inflammasome activation is involved in the pathogenesis of CLD. Most strikingly, knowing about the functions of the NLRP3 inflammasome allows us more effectively to provide novel insights in inflammasome-based therapies by creating methods and agents. Some inhibitors of the NLRP3 inflammasome have already been processed in clinical research in phase 1 and phase 2 trials in various subjects including healthy volunteers and patients with CLD. Although the current work provides insight of therapeutic potential for CLD by regulating the NLRP3 inflammasome signaling many questions remain to be addressed conclusively and new questions have arisen. For instance, The NLRP3 inflammasome can be activated in KCs, hepatocytes, HSCs, and intestinal epithelial cells in the development of CLD. However, the mechanism underlying the potential interaction of NLRP3 inflammasome activation in these cells in the liver remains unclarified. Furthermore, since the availability of rodent models could not exhibit the entire phenotypic spectrum of clinical CLD, several conflicting results in animal models have been reported. Thus, future developments will be crucially dependent upon understanding the underlying mechanisms of NLRP3 inflammasome activation by various exogenous and endogenous danger signals. Clearly, research into the regulation and function of the NLRP3 inflammasome in the pathogenesis of CLD will remain an exciting area that will continue to challenge our understanding of inflammasome biology. In the future it is anticipated that the NLRP3 inflammasome signaling cascade can be helpful in therapeutic strategies against CLD.

## Author Contributions

XW designed the subject of this review article, drafted the manuscript, and prepared the figures and tables. LD helped to revise the manuscript and figures. XL and JL gave the constructive comments and critically reviewed the manuscript. All authors read and approved the final manuscript.

## Conflict of Interest Statement

The authors declare no commercial or competing financial relationships that could be construed as a potential conflict of interest.

## Abbreviations

Casp, caspase; NOD, nucleotide-binding oligomerization domain; NLRs, NOD-like receptors; NLRP3, NLR family pyrin domain containing 3; ASC, apoptosis-associated speck-like protein containing a casp-recruitment domain; IL-1β, interleukin-1β; CLD, chronic liver disease; LF, liver fibrosis; HCC, hepatocellular carcinoma; HBV, hepatitis virus B; HCV, hepatitis virus C; KCs, Kupffer cells; CHC, chronic hepatitis C; NASH, non-alcoholic steatohepatitis; HSCs, hepatic stellate cells; NF-κB, nuclear factor kappa B; TLRs, Toll-like receptors; CHB, chronic hepatitis B; ALP, alkaline phosphatase; ATP, adenosine triphosphate; ALT, alanine aminotransferase; AST, aspartate aminotransferase; IFN, interferon; NAFLD, non-alcoholic fatty liver disease; MCD, methionine-choline-deficient; FAs, fatty acids; ALD, alcoholic liver disease; NKT, natural killer T; ILG, isoliquiritigenin; SFN, sulforaphane; BMDMs, bone marrow-derived macrophages; α-SMA, α-smooth muscle actin.
